# Identification and expression analysis of bZIP transcription factors in *Setaria italica* in response to dehydration stress

**DOI:** 10.3389/fgene.2024.1466486

**Published:** 2024-08-30

**Authors:** Xuefei Yang, Changyong Gao, Yaqian Hu, Qianru Ma, Zejun Li, Jing Wang, Zhaoqun Li, Li Zhang, Dongming Li

**Affiliations:** ^1^ Key Laboratory of Herbage & Endemic Crop Biology, Ministry of Education, School of Life Sciences, Inner Mongolia Normal University, Hohhot, China; ^2^ College of Agriculture and Bioengineering, Heze University, Heze, China; ^3^ College of Agriculture, Shanxi Agricultural University, Taigu, China

**Keywords:** foxtail millet, bZIP transcription factor, dehydration stress, gene expression, transcriptome

## Abstract

Among the largest transcription factor families in plants, bZIPs are crucial for various developmental and physiological processes, particularly abiotic stress resistance. *Setaria italica* has become a model for understanding stress resistance mechanisms. In this study, we identified 90 bZIP transcription factors in the *Setaria italica* genome. *SibZIPs* were classified into 13 groups based on references to *Arabidopsis* bZIPs. Members in the same group shared similar motifs and gene structure pattern. In addition, gene duplication analysis indenfied 37 pairs of segmental duplicated genes and none tandem duplicated genes in *S. italica* suggesting segmental duplication contributed to the expansion of the *S. italica* bZIP gene family. Moreover, the number of *SibZIPs* genes (39) exhibiting higher expression in roots was significantly more than that in other organs. Twelve *SibZIP* genes were upregulated in response to dehydration stress. In conclusion, our study advances the current understanding of *SibZIP* genes and provide a number of candidates for functional analysis of drought tolerance in *S. italica*.

## 1 Introduction

Drought stress is a major abiotic stress that affects global crop productivity, with almost all important crops being highly sensitive to drought (Zandalinas et al., 2018). To maintain normal growth and development under dehydration stress, plants have developed adaptive regulatory mechanisms to increase drought tolerance (Gupta et al., 2020). One such mechanism is the use of transcription factors (TFs) to regulate (inhibit or activate) specific protein expression to generate appropriate responses (Baillo et al., 2019; Hrmova and Hussain, 2021). The basic leucine zipper motif (bZIP) is a major TF family that actively responds to dehydration stress (Joo et al., 2021).

The bZIP TFs have 60–80 amino acid (aa) residues, with a basic structure comprising an alkaline binding domain and a leucine zipper dimerization motif (Hurst, 1995). The former contains a relatively conserved ACGT core motif in the form of DNA cis-elements, such as the G Box (CACGTG), C Box (CACGTC), and A Box (TACGTA) (Izawa et al., 1993; Foster et al., 1994). The latter is composed of two typical α-helices, each with at least four leucines (Leu) or another hydrophobic residue (e.g., isoleucine, valine, methionine) at every seventh position.

Members of the bZIP family play important regulatory roles in seed maturation (Alonso et al., 2009), flower development (Abe et al., 2005), carbon and nitrogen metabolism (Baena-González et al., 2007), and abiotic stress responses (Uno et al., 2000). Numerous studies have shown that plant could withstand drought stress by bZIP TFs through abscisic acid (ABA)-dependent pathways (Hrmova and Hussain, 2021; Guo et al., 2024). ABF1, AREB1/ABF2, ABF3, and AREB2/ABF4 could enhance drought stress tolerance in *Arabidpsis* (Yoshida et al., 2010; Yoshida et al., 2014). The manipulation of *AREB1*(*AtbZIP36*), which is involved in ABA response pathway, has been shown to improve drought tolerance in *Arabidpsis* (Fujita et al., 2005). Similarly, *OsABF1* (*OsbZIP42)* acts as a positive regulator of drought tolerance in rice (Joo et al., 2019). Moreover, *OsbZIP23, OsbZIP45, OsbZIP46* and *OsbZIP72* also play important roles in drought tolerance in rice (Park et al., 2015; Tang et al., 2012; Lu et al., 2009). In addition, bZIP TFs in other plant species such as *GmbZIP2* in soybean, *ZmbZIP76* in maize, and *PtrbZIP3* in Populus trichocarpa are also involved in drought tolerance (Yang et al., 2020; He et al., 2024; Zhou et al., 2023).

Initial genome-wide analyses of bZIP family members were made possible through the availability of genome sequences from the model plants *Arabidopsis* and rice (*Oryza sativa*). *Arabidopsis* bZIP proteins are classified into 10 groups and one unclassified group based on phylogeny and conserved motifs (Jakoby, 2002). In rice, the majority of phylogenetically related bZIP proteins were found to have similar DNA-binding properties based on binding site analyses (Nijhawan et al., 2008). As more plant genomes have been sequenced, bZIP families have been characterized in maize (Wei et al., 2012), sorghum (Wang et al., 2011), wheat (Agarwal et al., 2019), soybean (Zhang et al., 2018), barley (Pourabed et al., 2015), peanut (Wang et al., 2019), and peach (Aslam et al., 2023).

A draf genome of Setaria was developed over a decade ago, and the genome sequence has been updated to version 2.2 at Phytozome database (Zhang et al., 2012). Enomous trancriptomic and proteomic data of Setaria under drought stress has been generated (Pan et al., 2018; Zhang et al., 2022; Gao et al., 2023), and investigation of these data using bioinformatic and biotechnology approaches help us identify the possible candidate genes of drought tolerance. In this study, we used foxtail millet (*Setaria italica* L.) as a model species for examining the role of bZIP in drought tolerance. We performed a genome-wide analysis of the bZIP gene family in the *S. italica* genome to identify and classify *S. italica* bZIP (*SibZIP*) genes. We also analyzed *SibZIP* expression profiles within different organs and under drought treatment. The results from our study should offer valuable information for further understanding the role of *SibZIPs* in drought tolerance.

## 2 Materials and methods

### 2.1 Identification of the bZIP gene family in *Setaria italica*

To identify bZIP genes in *S. italica*, the bZIP domain (PF00170) was downloaded from the Pfam website (http://pfam.xfam.org/). HMMER software was then used to screen the protein sequences of *S. italica*, with a threshold set at an E-value <10^−5^. In addition, 78 Arabidosis AtbZIPs and 89 rice OsbZIP protein sequences, download from TAIR (http://arabidopsis.org) and TIGR (http://www.tigr.org) respectively, were used as queries to search *S. italica* protein sequences. Subsequently, the candidate proteins were further screened, and the conserved domains were validated using SMART (http://smart.embl-heidelberg.de/) in combination with the NCBI CDD online analysis website (https://www.ncbi.nlm.nih.gov/Structure/cdd/wrpsb.cgi). The incomplete domain and redundant protein sequences were manually removed. Finally, a total of 90 *SibZIP* genes were identified from *S. italica* (Supplementary Table S1).

### 2.2 Chromosomal localization and syntenic analysis of *SibZIP* genes

The genome annotation file (Gene Transfer Format/General Feature Format version 3 [GTF/GFF3]) of *S. italica* was downloaded from the Phytozome database (https://phytozome-next.jgi.doe.gov/), and the chromosomal localization of each *SibZIP* gene was displayed using TBtools (Chen et al., 2020).

The gene duplication of the *SibZIP* genes in *S. italica* was predicted using MCScanX (Wang et al., 2012). The syntenic relationships between *SibZIP* genes and *bZIP* genes from *A. thaliana* and *O. sativa* were visualized by TBtools. The nonsynonymous substitution rate (Ka), synonymous substitution rate (Ks), and Ka/Ks ratio were determined using TBtools (Supplementary Table S2, S3, S4). The selection pressure of duplicated genes was evaluated using the Ka/Ks ratio. Ka/Ks > 1 suggests positive selection, Ka/Ks = 1 suggests neutral selection, and Ka/Ks < 1 meant negative selection (Krishnamurthy et al., 2015).

### 2.3 Phylogenetic tree construction of SibZIP proteins

The full bZIP protein sequences of 13 AtbZIP proteins from *Arabidopsis,* 89 *OsbZIP* proteins from rice and 90 SibZIP proteins were subjected to multiple sequence alignment using the MUSCLE wrapper and trimAL wrapper of TBtools (Supplementary Table S5). The results were used to construct a neighbor-joining phylogenetic tree in TBtools. Bootstrap values were calculated with 1,000 iterations. The phylogenetic tree was embellished using iTOL v6.7.6 (https://itol.embl.de/).

### 2.4 Conserved domains and gene structure analysis of the *SibZIP* genes

Protein motifs were identified using Multiple Expectation Maximization for Motif Elicitation (MEME) (http://meme.nbcr.net/meme/). The analysis was performed with the following settings: number of repetitions, any; maximum number of motifs, 20; and optimum width motifs, 10–60.

Full bZIP protein sequences from *S. italica* were subjected to the Conserved Domain Database (CDD) from the National Center for Biotechnology Information (NCBI), and the results were used to construct gene structure photographs using Gene Structure View in TBtools.

### 2.5 In silico expression profiling of *SibZIP* genes

The gene expression profiling data of *SibZIP* genes were retrieved from the Multi-omics Database for *S*. *italica* (MDSi) (http://foxtail-millet.biocloud.net/home). The reads per kilobase per million (RPKM) was downloaded (Supplementary Table S6) and a heatmap was generated in the HeatMap in TBtools.

In addition, transcriptomic data from roots of two drought-tolerant cultivars (Ci328 and Ci409) under normal condition (ERX5299071 and ERX5299091) and drought condition (ERX5987296 and ERX5299098) were retrieved from the European Nucleotide Archive (https://www.ebi.ac.uk/ena, PRJEB43702). The expression data of *SibZIP* genes were normalized as Log_RPKM_ (Supplementary Table S7) and the expression levels of the *SibZIP* genes were visualized using HeatMap in TBtools.

### 2.6 Plant materials and drought treatment

Seeds of ‘Yugu1’ were obtained from Inner Mongolia Agriculture University, Hohhot, China, and grown in a greenhouse under the following conditions: 20 h of light (150 μmol⋅m^-2^⋅sec^-1^) at 26°C ± 2°C and 4 h of darkness at 22°C ± 2°C. eight pots containing 5-week-old seedlings were under drought stress for 10 days and re-watering afterwords. The pots with normal watering were used as controls. Roots from seedlings under drought stress and re-watering were collected and then stored at −80°C until RNA isolation. Three independent replicates were carried.

### 2.7 *SibZIPs* gene expression in response to dehydration stress

Total RNA was extracted from roots using RNAiso Plus (TaKaRa, T9108) according to the protocol. The integrity of the extracted RNA was determined through 1.5% agarose gel electrophoresis and the quantity was measured with a NanoDrop. The cDNA was synthesized using PrimeScriptTM Ⅱfirst Strand cDNA Synthesis Kit (TaKaRa, 6210A). RT-qPCR was carried out using PrimeScript RT reagent Kit with gDNA Eraser (Takara, RR047A) on ABI7500 (Applied Biosystems, USA). The primers of *SibZIP* genes were designed by Primer Premier 5.0 (Supplementary Table S8). The fold change of the expression levels of *SibZIP* genes was calculated via relative quantification (2^-△△CT^) and *SiACTIN* was used as internal reference gene (Zhang et al., 2017).

## 3 Results

### 3.1 Identification of *SibZIP* genes

An initial search of the Phytozome database yielded 92 *SibZIP* members. Of these, 2 were redundant transcripts, and the remainder contained 90 putative *SibZIP* genes, which were named *SibZIP1*-*SibZIP90* based on their chromosomal position (Supplementary Table S1).

The predicted size of the SibZIP proteins was 319 aa on average, but they spanned a wide range (132–759 aa). Approximately 50.5% of the SibZIP proteins had predicted sizes between 200 and 400 aa, 27.5% had sizes less than 200 aa, and 23% had sizes greater than 400 aa. The longest predicted protein sequence (759 aa) was SibZIP62, whereas the shortest (132aa) was SibZIP69. The isoelectric point (pI) values (4.69–11.97) and molecular weights (15.260–80.305 kDa) of the SibZIPs varied widely. Supplementary Table S1 provides further characteristics of the SibZIP proteins.

### 3.2 Chromosome localization, gene duplication and syntenic analysis of *SibZIP* genes

**FIGURE 1:**
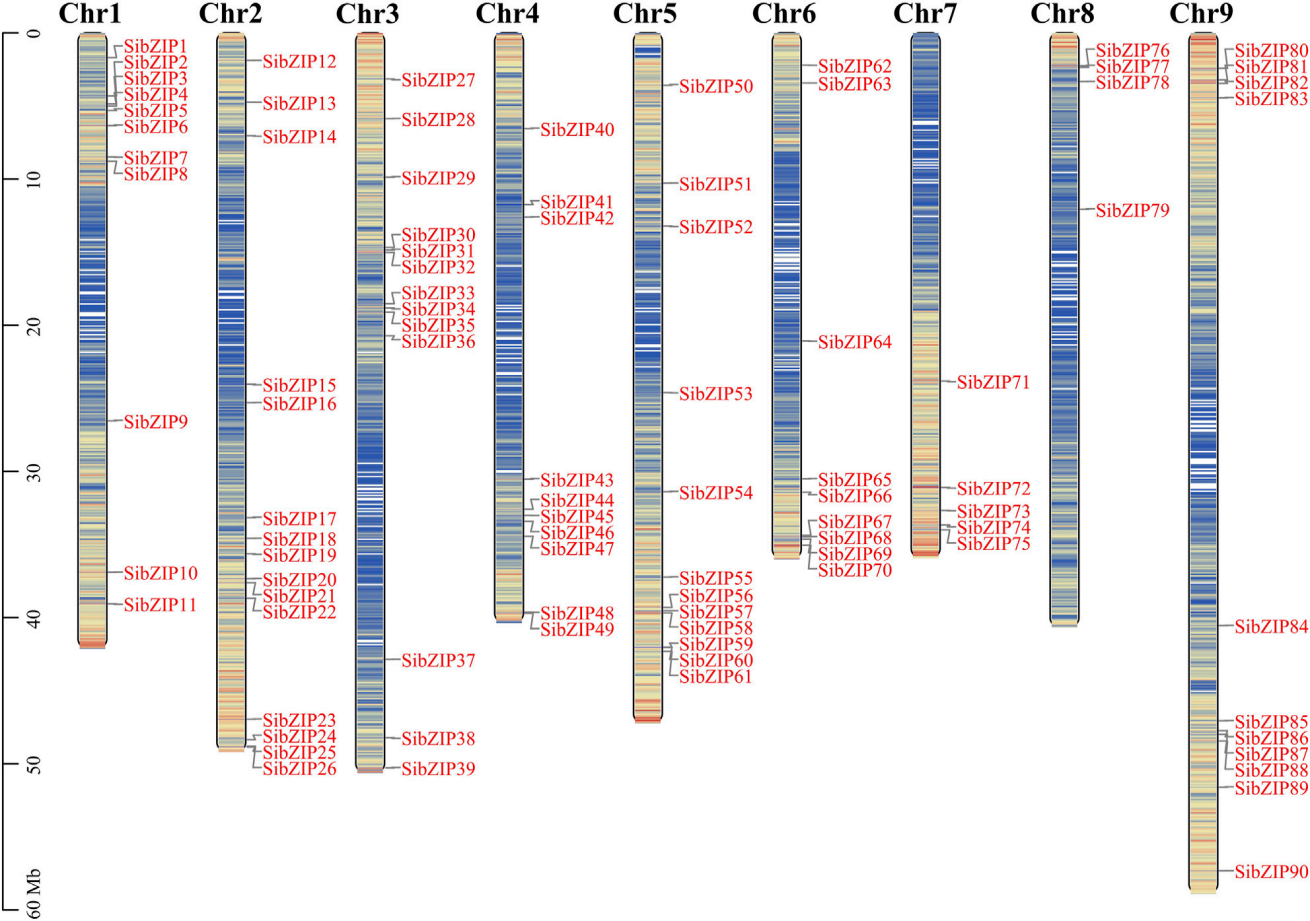
Distribution of 90 *SibZIP* genes onto nine *Setaria italica* chromosomes. Graphical representation of physical locations for each *SibZIP* gene on *Setaria italica* chromosomes (numbered Chr1–9). Chromosomal distances are given in Mb.

The 90 *SibZIP* genes were unevenly distributed across the 9 chromosomes of *S. italica* (Figure 1). The number of genes on each chromosome was unrelated to chromosome size. Chromosome 2 had the greatest number of genes (15), accounting for 16.7% of all *SibZIP* genes, while chromosome 8 contained the least number of genes (4, 4.4%).

**FIGURE 2:**
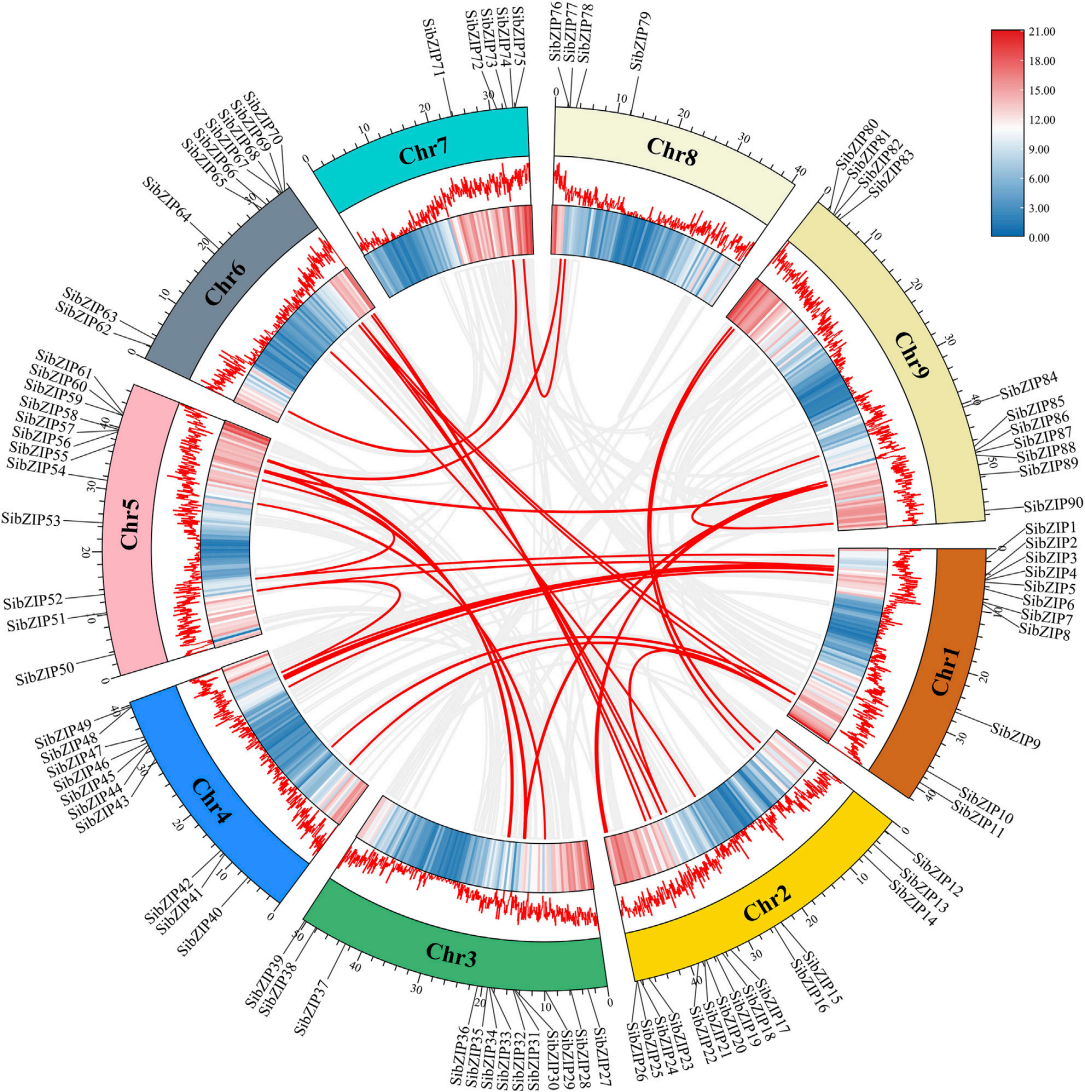
Gene duplication examination of *SibZIP* genes.

Moreover, gene duplication of the *SibZIP* genes was predicted using MCScanX. Thirty-seven segmental duplicated gene pairs were detected on different chromosomes of *S. italica,* and no tandemly duplicated genes were detected (Figure 2; Supplementary Table S2). This result indicates that segmental duplication contributed to the expansion of the *S. italica* bZIP gene family during evolution. The Ka/Ks ratio of 37 pairs of segmental duplicated genes were all lower than 1, varying from 0.07 to 0.90. The Ka/Ks value of *SibZIP21* and *SibZIP67* is larger than 0.9 and the ratios of the rest gene pairs were all less than 0.5.

**FIGURE 3:**
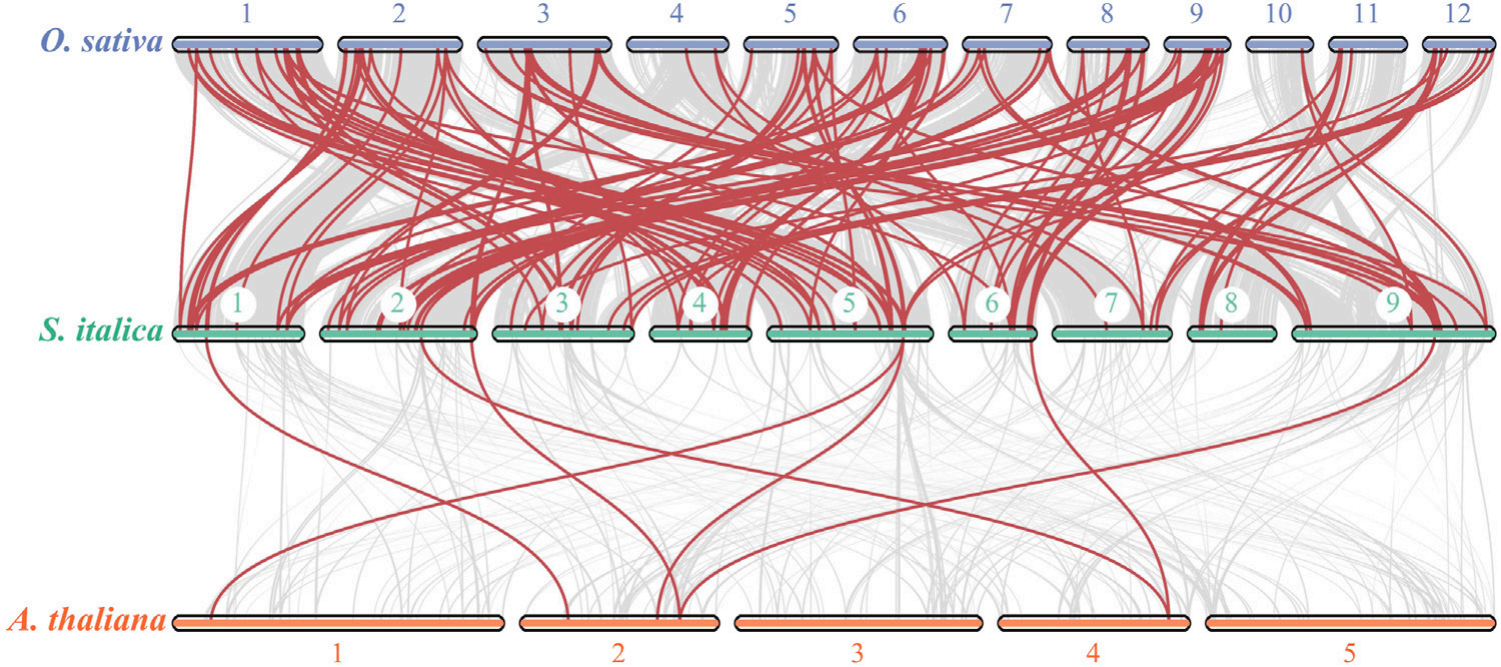
Synteny of bZIP gene family members between *Setaria italica*, *A. thaliana*, and *Oryza sativa*.

In addition, to investigate the evolutionary relationships of bZIP genes from *S. italica*, *A. thaliana* and *O. sativa*, syntenic analysis was conducted using TBtools. The results revealed that 84 *SibZIP* genes exhibited collinear relationships with rice *OsbZIP* genes, while only 7 *SibZIP* genes exhibited collinear relationships with *Arabidopsis AtbZIP* genes (Figure 3; Supplementary Table S3, S4).

### 3.3 Phylogeny, motif and gene structure analysis of the *SibZIP* genes

**FIGURE 4:**
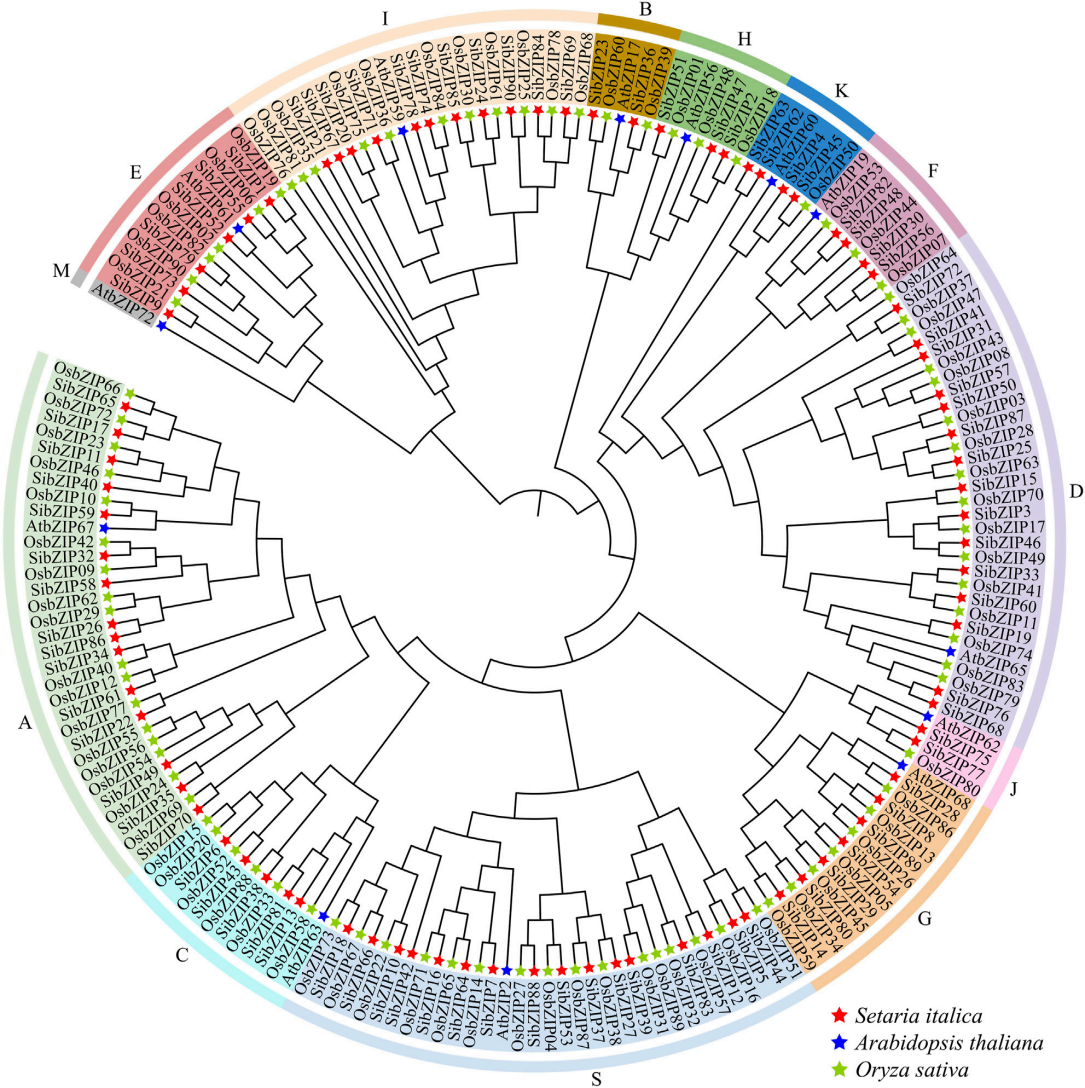
Phylogenetic tree of bZIP proteins from *Setaria italica* (*SibZIPs*) and *Arabidopsis* (AtbZIPs) and *O. sativa* (OsbZIPs). The proteins were classified into 13 distinct clusters. Each group was assigned a different color. The name of groups (A, B, C, D, E, F, G, H, I, J, K, M and S) were shown at the outside of the circle.

Based on the multiple sequence alignment and the previously reported *AtbZIP* classification, the *SibZIP* genes and rice *OsbZIP* genes were assigned to 13 groups (A-M, and S) (Figure 4). The larger group was Group S containing 17 *SibZIPs*, followed by Group A (15) and Group D (15). The smallest groups were B and J with only two genes. In addition, no *SibZIP* and *OsbZIP* were classified wtih *Arabidopsis AtbZIP72* (Group M).

**FIGURE 5:**
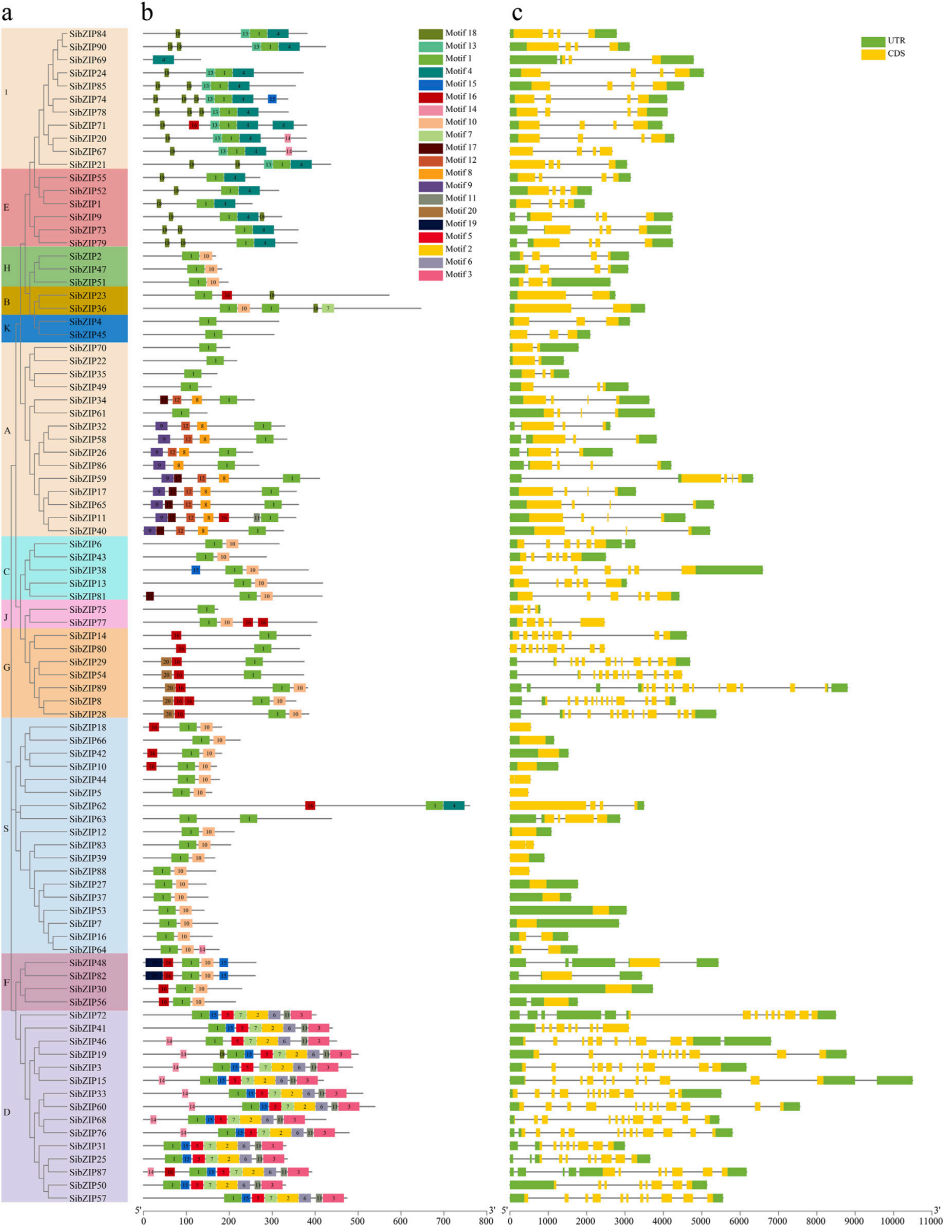
Schematic diagram of amino acid motifs and gene structure of *SibZIP* genes from different groups. **(A)** Phylogenetic tree of SibZIP genes. **(B)** Schematic diagram of amino acid motifs of SibZIP genes from different groups. **(C)** Schematic diagram of gene structure of SibZIP genes from different groups. Motif analysis was performed using Tbtools software as described in the Methods. The black solid line represents the corresponding *SibZIP* genes and its length. The different-colored boxes represent different motifs and their position in each *SibZIP* sequence.

We identified 20 motifs in the 90 *SibZIP* genes and mapped their distribution to the phylogenetic tree (Figures 5A,B; Supplementary Table S9). All *SibZIPs* contained the basic leucine zipper domain (Motif 1; Supplementary Figure S1). Group A contained specific motif 8 (RQGSLGSLTLEEFLVRLGVVREDMGSD), which contains a phosphorylation site RXXS/T (Uno et al., 2000). Group D contained specific motif 3 and motif 6 which are glutamine rich (Q-rich) domains at C-terminus. Motif 13 was observed only in Group I, while motif 20 was only present in Group G. In addition, some motifs were present in multiple groups. Groups E and I, for instance, both contained motifs 4 and 18, whereas motif 10 was shared across most groups except D. Together, these observations demonstrate that most *SibZIPs* in the same group also tended to contain similar motifs.

The distributions of the coding sequences (CDSs), untranslated regions (UTRs), and introns of the *SibZIPs* are displayed in Figure 5C. The number of introns ranged from 1 to 14, and the number of introns in the same subgroup was similar. Most *SibZIP* genes from Group S were intronless.

### 3.4 Tissue-specific expression analysis of *SibZIP* genes

**FIGURE 6:**
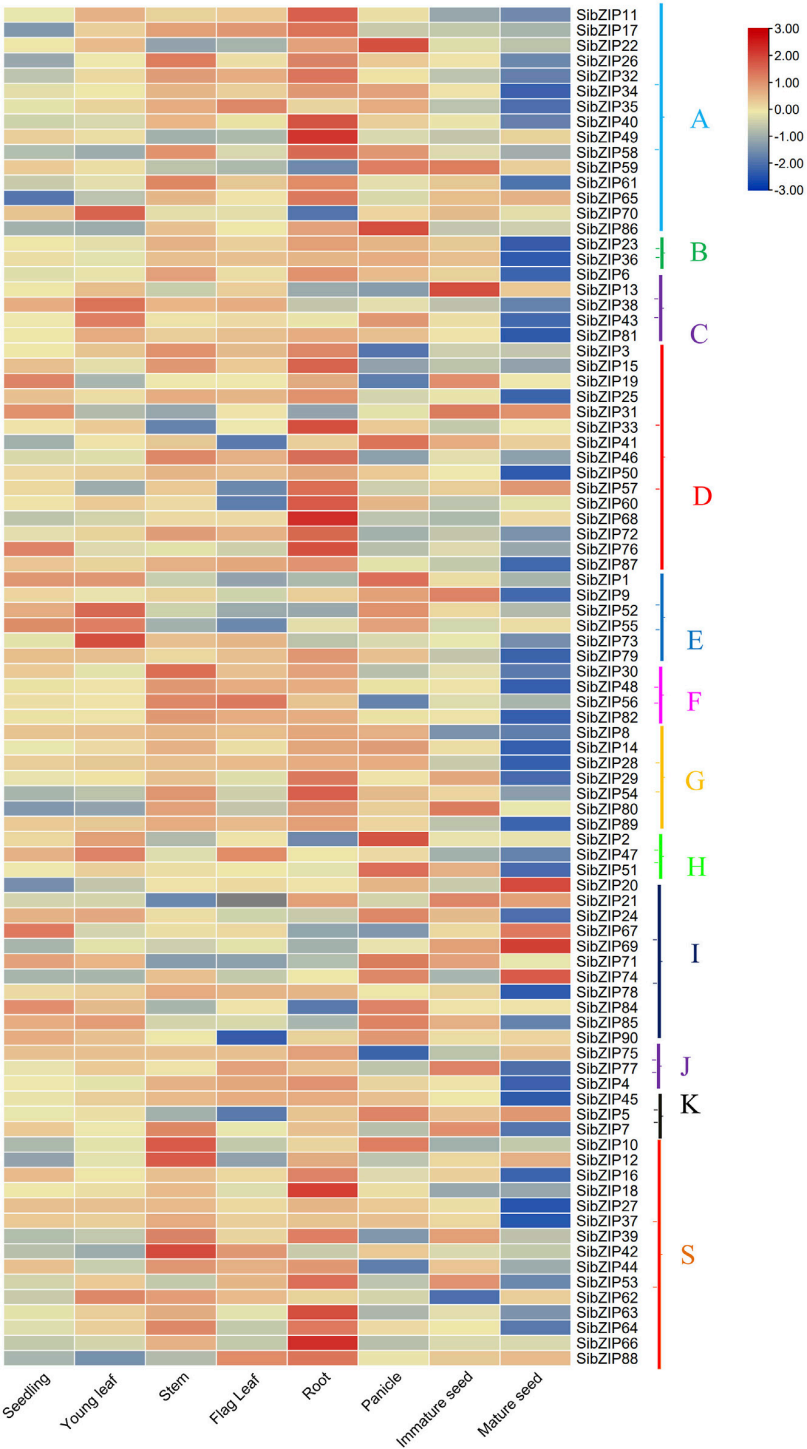
Heat-map showing the expression pattern of *SibZIP* genes in eight tissues namely seedlings, young leaves, stems, flag leaves, roots, panicles, immature seeds and mature seeds. The heat-map shows gene expression of *SibZIP* genes in different groups (A, B, C, D, E, F, G, H, I, J, K and S). The color scales for fold-change values are shown at the right. The figure showed that most *SibZIP* genes were highly expressed in at least one of the tested tissues. Note that expression values mapped to a color gradient from low (blue) to high expression (orange).

The gene expression profiling data of *SibZIP* genes from eight different organs (including seedling, young leaf, stem, flag leaf, root, panicle, immature seed and mature seed) were retrieved from the MDSi database (Supplementary Table S6). A heatmap was generated using TBtools (Figure 6), which showed that seven *SibZIPs* (*SibZIP20, SibZIP57, SibZIP58, SibZIP65, SibZIP69, SibZIP73* and *SibZIP74*) were expressed in all the tissues, but many genes were specific to certain organs (Figure 6; Supplementary Table S6). For example, 39 *SibZIP* genes have highest expression in roots, and 13 and 14 genes were in panicle and stem. *SibZIP12* were only expressed in mature seeds.

### 3.5 *SibZIP* gene expression patterns under dehydration stress

**FIGURE 7:**
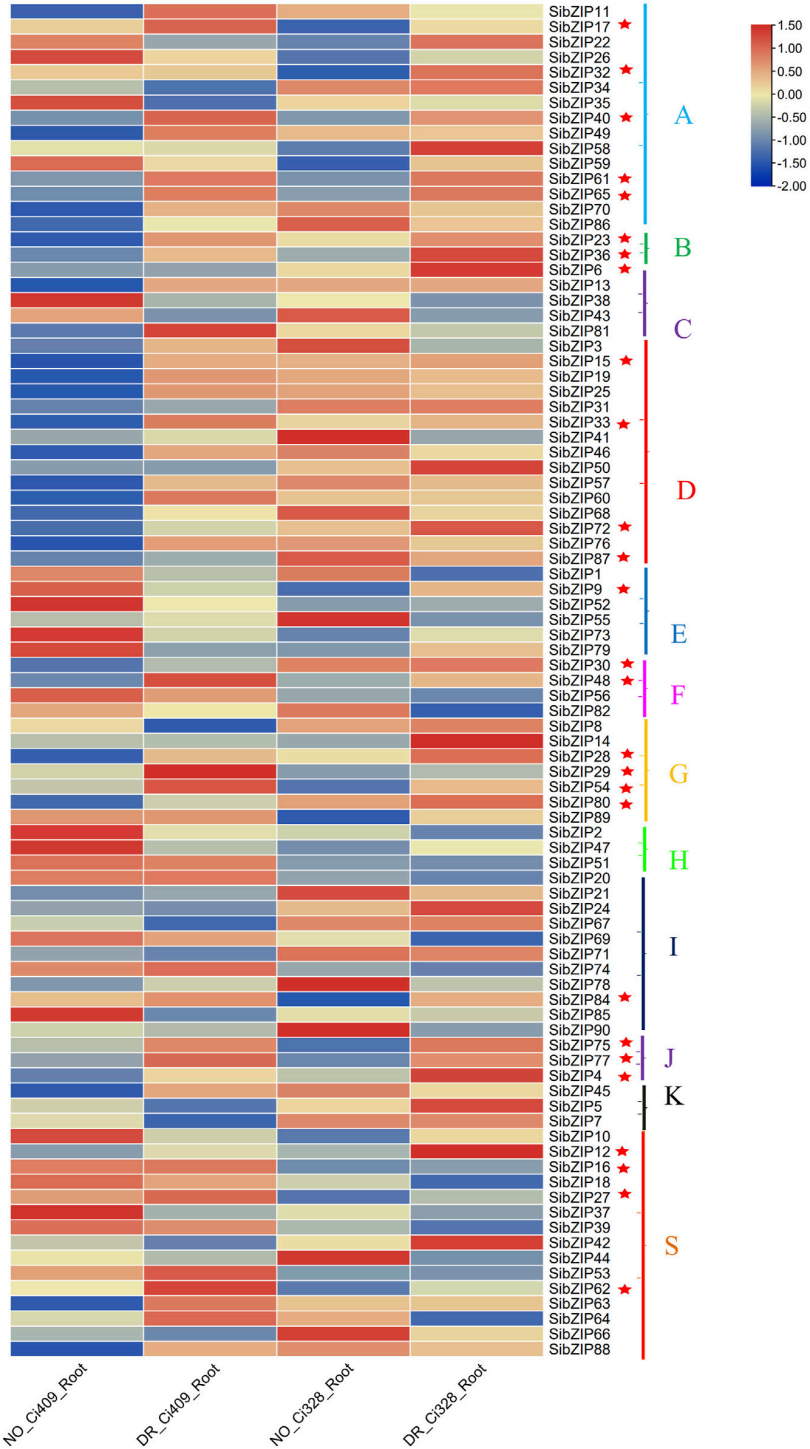
*SibZIP* genes expression patterns under dehydration stress. The transcriptome data of tolerance cultivars (Ci328 and Ci409) under normal condition and drought stress condition was downloaded to investigate *SibZIP* gene expression patterns under dehydration stress (Zhang et al., 2022).The heat-map shows gene expression of *SibZIP* genes in different groups (A, B, C, D, E, F, G, H, I, J, K and S).Twenty seven SibZIP genes were up-regulated after drought stress in both Ci328 and Ci409 and marked with asterisks.

The transcriptome data of tolerance cultivars (Ci328 and Ci409) under drought stress was used to investigate *SibZIP* gene expression patterns under dehydration stress. The expression patterns of the *SibZIP* genes in roots under drought treatment and under normal conditions are shown in the heatmap (Figure 7; Supplementary Table S7). The results showed that 27 *SibZIP* genes were upregulated after drought stress in both Ci328 and Ci409 and marked with asterisks in Figure 7. These genes were from different groups. Group A had highest number of upregulated genes (5 genes), followed by Group D (4) and Group G (4).

**FIGURE 8:**
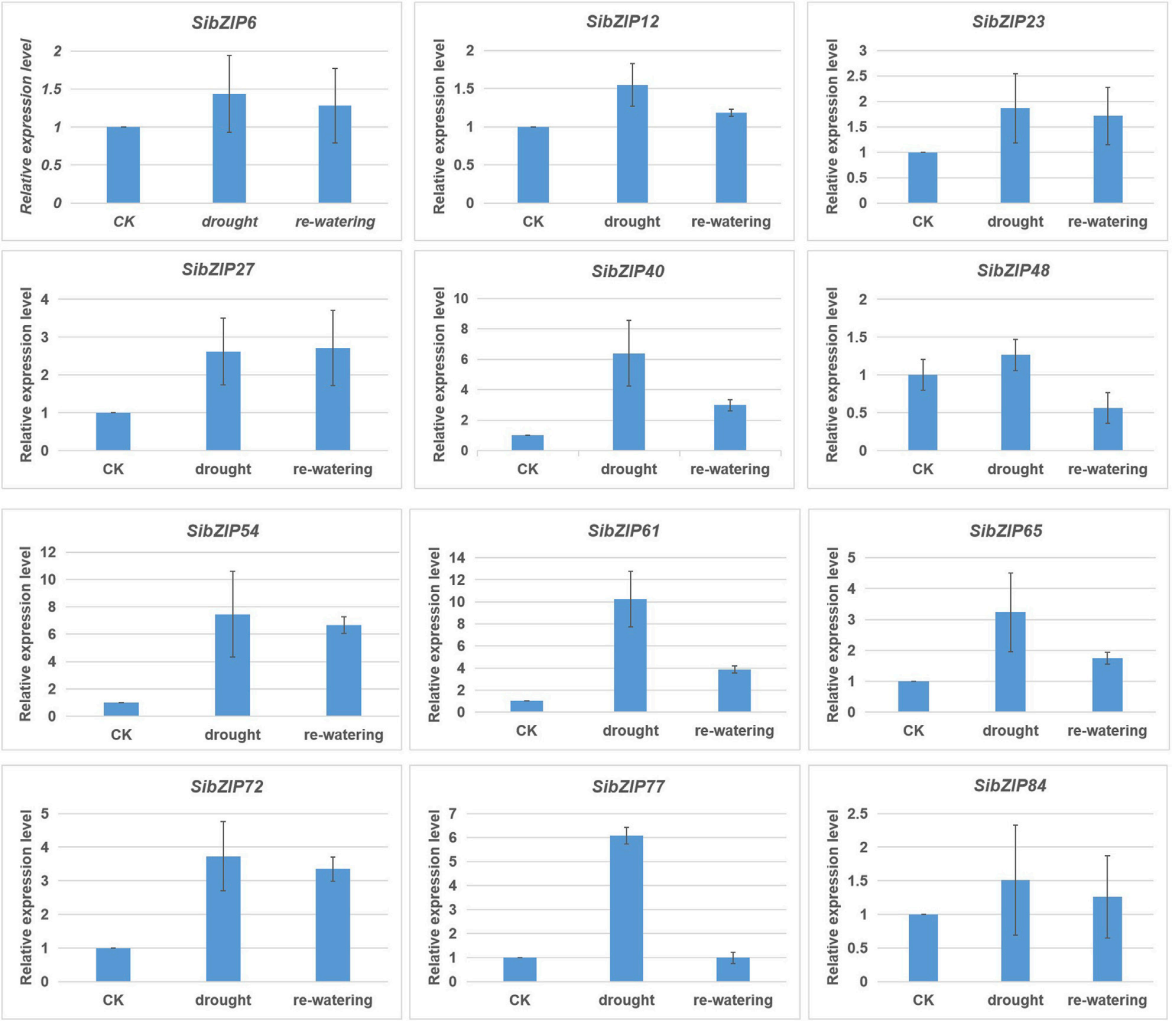
The RT-qPCR expression analysis of *SibZIP* genes under dehydration stress.

In addition, RT-qPCR were used to confirm the expression patterns of 12 genes (*SibZIP6*, *SibZIP12*, *SibZIP23*, *SibZIP27*, *SibZIP40*, *SibZIP48*, *SibZIP54*, *SibZIP61*, *SibZIP65*, *SibZIP72*, *SibZIP77* and *SibZIP84*). The RT-qPCR profiles were shown in Figure 8. The results showed that 12 *SibZIP* genes were all upregulated after seedlings were subjected to drought stress, and decreased after re-watering (Figure 8). Additionally, over 5 fold-increase was observed for *SibZIP40*, *SibZIP54*, *SibZIP61* and *SibZIP77*.

## 4 Discussion

*S. italica* is an important grain and forage crop in China, known for its excellent drought and barren soil tolerance (Muthamilarasan and Prasad 2015). In recent years, due to the impact of climate change, the cultivation of crops in arid regions has been receiving increasing attention (Zandalinas et al., 2018). Therefore, elucidation of the drought tolerance mechanisms in *S. italica* is of significant importance. Drought is a complex trait and regulated by many genes from different pathways, transcription factors including bZIP transcription factors actively respond to dehydration stress (Baillo et al., 2019; Joo et al., 2021).

In this study, we successfully identified 90 *SibZIPs* genes in *S. italica* using an updated *S. italica* genome at Phytozome database and added 17 more *SibZIPs* genes than previous identification of bZIP genes in *S. italica* (Liu et al., 2016). Phylogenetic analysis, conserved motif and gene structure analysis demonstrated that *SibZIPs* can be categorized into 13 distinct groups, with members of the same groups sharing similar motifs and gene structure pattern. Seventy five *Arabidopsis* bZIPs was first classified into 10 groups (A-I, and S) and Dröge-Laser proposed a updated classification of 13 groups (A-M, and S) (Jakoby et al., 2002; Dröge-Laser et al., 2018). The number of bZIP family members and classification in different species was diverse. For instance, 89 rice OsbZIP proteins were classified into 10 clades and 125 maize bZIPs were identified into 11 groups (Wei et al., 2012). Moreover, 98 pearl millet *PgbZIPs* into 12 subfamilies (Jha et al., 2024), and 86 poplar bZIP genes into 12 subfamilies (Zhao et al., 2021). Although the groups differed slightly in their the number of classification, subfamilies across different plants shared common 10 subfamilies (A-I, and S).

Gene duplication is the major force of bzip gene family expansion (Corrêa et al., 2008). We indenfied 37 pairs of segmental duplicated genes and none tandem duplicated genes in *S. italica*. This result indicates that segmental duplication contributed to the expansion of the *S. italica* bZIP gene family during evolution. Our result is generally in line with previous studies on the bZIP family in cucumber (Baloglu et al., 2014), legume (Wang et al., 2015), poplar (Zhao et al., 2021), tobacco (Li et al., 2021) Solanum tuberosum (Herath and Verchot, 2020), *Perilla frutescens* (Huang et al., 2024), ect. In addition, The Ka/Ks ratio of 37 pairs of segmental duplicated genes were all lower than 1, varying from 0.07 to 0.90. The ratios of most gene pairs were all less than 0.5. This result suggested that the duplicated genes was under negative selection and exhibited little functional divergence (Krishnamurthy et al., 2015).

In silico expression profiling in eight organs of *S. italica* revealed that seven *SibZIP* genes were ubiquitously expressed in all the tissues, suggesting transcriptional regulation of a broad gene set. Moreover the number of *SibZIPs* genes (39, 43%) exhibiting higher expression in roots was significantly more than that in other organs, which was also observed in cassava (Hu et al., 2016). In addition a number of transcriptome studies of Setaria italica in response to drought stress have generated large amount of genomic data (Yi et al., 2022; Wang et al., 2023; Zhang et al., 2022). The transcriptome data of tolerance cultivars (Ci328 and Ci409) under drought stress was used to investigate *SibZIP* gene expression patterns under dehydration stress in this study. Interestingly, most of *SibZIPs* genes showed response to drought stress in each drought tolerant cultivar, but only 27 genes showed response in two drought tolerant cultivars. In addition, 12 out of 27 genes were further investigated on their expression under drought stress using RT-qPCR and they were all upregulated after drought stress, indicating their possible function in drought tolerance. Numerous bZIP TFs have been identified as positive regulators of drought stress. For example, *OsbZIP23* homologous genes of *SibZIP40*, are involved in ABA-dependent drought regulation (Park et al., 2015). *OsbZIP*72 overexpression significantly improved drought tolerance and ABA sensitivity in rice (Lu et al., 2009). Moreover, a role for *GmTGA17* in the drought and salt tolerance was also suggested by the upregulation of *GmTGA17* in both *Arabidopsis* and soybean (Li et al., 2019).

## 5 Conclusion

In this study, we identified and characterized bZIP TFs in *S. italica* using bioinformatics analysis and transcriptome sequencing data. Through a phylogenetic analysis, our genome-wide analysis revealed 90 *SibZIP* genes that were subsequently classified into 13 groups (with reference to *Arabidopsis* bZIP classification). The analysis of 20 conserved motifs and gene structure analysis supported this classification. Moreover, transcriptome data and RT-qPCR analysis revealed a number of *SibZIP* genes were upregulated under drought stress. This comprehensive study on *S. italica* bZIPs under drought stress provides useful information for further investigating the molecular mechanism of plant adaptation to drought stress.

## Data Availability

The original contributions presented in the study are included in the article/Supplementary Material, further inquiries can be directed to the corresponding authors.
