# Supplementary figures and images for: Identification and expression analysis of bZIP transcription factors in Setaria italica in response to dehydration stress
Source: Front Genet. 2024 Aug 30;15:1466486. doi: 10.3389/fgene.2024.1466486 (PMC11392892; doi:10.3389/fgene.2024.1466486)

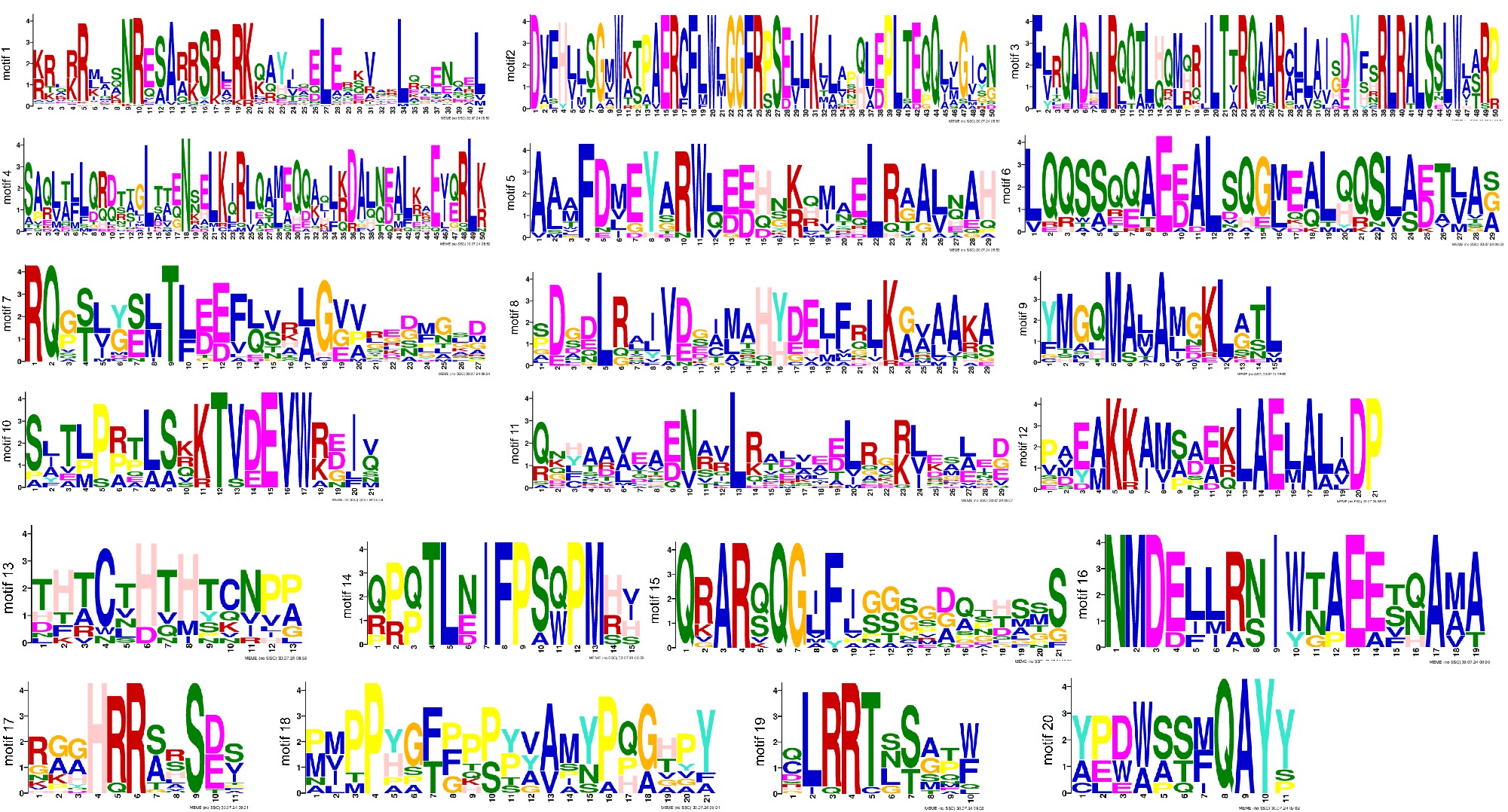

Supplement: Supplementary file 1 [file Image1.JPEG]
